# Microscopic

**Published:** 1880-01

**Authors:** S. O. Gleason


### MICROSCOPIC.

S. O. GLEASON, M. D.

The microscope is the great *revelator* of modern times. It is not to be regarded with contempt, because it deals with things invisible to the naked eye. The scientific results of recent investigation are *immense.* They reach out with the welfare of all human life. Multitudes of minute organisms swarm in all the waters of our pools, streams and rivers. These can only be seen by the aid of the microscope. They not only deeply interest the observer, but they play an important part in the great economy of life in this world. The human race is more dependent upon these unseen things, than is generally known. They keep our stagnant pools, in a great measure from being terrific hot beds of pestilence and disease. The various forms of microscopic vegetable and animal life that lives in those waters, do for the purity of the water, what animals and trees do for the air we breathe. The vast numbers of minute vegetable forms give off oxygen, while the animal life gives off carborn. The one form helps the other to live, so that the water keeps comparatively harmless. There stands on my window sill, a glass globe containing a body of water and a few small fish. It has not been changed for months. I often take a few drops of this water and examine it under the microscope and find there multitudes of scavengers at work to keep the water pure, so that it will sustain the fish, a higher form of life. They furnish good food for the fish, and make the water fit for them to use. Here in this unseen world lies one of the grand, and sublime truths that pertain to the mystery of our life. The amount of food furnished for fish and other forms of life, by these microscopic organisms, is absolutely immense. There is then an interest to us, all in those little living things.

To the student of nature there is no field, so rich, so full of real interest as in that which the microscope gives us; absolutely a new world of life and beauty. What we have been accustomed to look on with almost contempt and disgust, namely, the stagnant pool, with the aid of the microscope, is revealed a rich harvest of choice treasures. Many forms of life having, as it were, glass shells, exqusitly carved, can be heated, the organic matter burned out, their little homes put on glass slips under cover, and kept as things of beauty, for an indefinite period of time.

Indeed, the little silicious caskets that once enclosed a form of life, have defied the march of time, and maybe found imbedded in the solid rock, in the chalk, in the clay, or deep down in the great ocean of waters. These little forms have thus built up large portions of the earths surface. To many of the forms now living, is attributed the fertility of the rice fields of the South.

Then, in those simple forms of life, we find the "gates ajar" to higher truths in the scientific research in the great field of all life, even the mysteries of our own organisms. So the student of the science of life, must begin with the lowest and minutest forms, in which life exists and study them as steps by which higher attainments become possible.

There are some forms of clay that are eaten by the lower orders of fish, and even much is consumed by what are called the dirt-eaters among mankind. The real nutrition of these kinds of earth, is due to the remains of our little microscopic animals. A large amount of this kind of deposit is found in Sweden. It is often mixed with flour, and used as bread. It is called mountain-meal. These shell-made deposits, are used in the South, in our own country, to a considerable extent.

As a matter of interest to the student, let us state that the delicate markings on the tiny shells, vary from 52.000 to 130.000 to the inch. Such minute work is almost inconceivable, yet it is absolutely true. Of this, more in future numbers of the BISTOURY.

